# Public Understanding of Risk and Benefit of Mifepristone: A Randomized Clinical Trial

**DOI:** 10.1001/jamanetworkopen.2024.60236

**Published:** 2025-02-06

**Authors:** Tamar Krishnamurti, Gianna White, Barry Dewitt, Elizabeth Mosley, Baruch Fischhoff

**Affiliations:** 1 Division of General Internal Medicine, University of Pittsburgh, Pennsylvania; 2 Department of Engineering and Public Policy, Carnegie Mellon University, Pittsburgh, Pennsylvania; 3 Keenan Research Centre for Biomedical Science, St Michael’s Hospital, Unity Health Toronto, Toronto, Ontario, Canada

## Abstract

This randomized clinical trial investigates whether a 1-page information sheet proposed by the US Food and Drug Administration (FDA) can provide patients with sufficient information to use mifepristone safely and effectively.

## Introduction

The US Food and Drug Administration (FDA) has proposed regulations requiring that a 1-page Patient Medication Information sheet (PMI) accompany all prescriptions. The goal is to provide “essential information that patients need” to use a drug safely and effectively.^1^

When evaluating drugs for regulatory approval, FDA balances estimates of the drug’s benefits and risks.^2^ Patients need those estimates when deciding whether the risks are acceptable, given the benefits. However, the proposed PMI does not require telling patients anything about benefits or about the estimated size of risks or benefits. Decision science research has found that people can understand and, critically, do use such estimates when clearly presented.^3^ Drawing on that research, we created a version of the PMI with quantitative risk and benefit information, which we compare with versions without that information. As a case study, we use the abortion and miscarriage management medication, mifepristone (Mifeprex), an increasingly used drug^4^ with risks and benefits that are widely misunderstood.^5^

## Methods

This randomized clinical trial’s protocol (Supplement 1) was approved by the Carnegie Mellon University institutional review board (NCT06320808). In March 2024, we used the Prolific online research survey service to recruit 330 female participants living in states with varied abortion access (eFigure 1 in Supplement 2 shows study flow). Participants provided electronic informed consent and received $10 for completing a 15-minute survey study (eAppendix in Supplement 2). We followed the Consolidated Standards of Reporting Trials (CONSORT) reporting guideline.

Participants were randomized to receive 1 of 3 forms: (1) the newly proposed 1-page FDA PMI; (2) a decision critical PMI, using the same format as FDA PMI, adding quantitative risk and benefit information, and (3) the multipage vendor communication provided by the pharmaceutical company to patients (eFigures 2-4 in Supplement 2). After viewing their assigned communication, participants answered questions assessing their comprehension of important information and the quality of the evidence.^6^ They also rated how easy the communication was to read and how useful it would be for deciding whether and how to take the drug. Kruskal-Wallis tests were used to evaluate overall differences, followed by Mann-Whitney *U* tests for comparing paired groups when overall differences were observed. A Bonferroni-correction accounted for multiple comparisons, α** = **.01, for ordinal variables (R Statistical Software version 4.3.2 [R Project for Statistical Computing]).

## Results

The final sample included 311 female participants with a mean (SD) age of 31.7 (7.1) years; 96 (30.9%) reported no more than a high school education (Table 1). The FDA and decision critical communications were similarly readable (mean [SD] FDA rating: 4.21 [0.71] vs decision critical: 4.25 [0.74]); both were superior to the vendor Communication (mean [SD] vendor rating, 3.63 [0.99]; *P* < .001 for both mean comparisons). Comprehension was significantly higher with the decision critical communication (6.34 [0.91]) than with either the FDA (5.40 [0.76]) or vendor communication (5.50 [1.17]) (*P* < .001 for both mean comparisons), as were ratings of how well the communication supported decision making around whether to use the drug (decision critical, 4.39 [0.67] vs FDA, 3.95 [0.85]; *P* < .001; and vs vendor, 4.13 [0.78]; *P* = .03) and how to use it (decision critical, 4.64 [0.59] vs FDA, 4.35 [0.63]; *P* < .001; and vs vendor, 4.33 [0.64]; *P* < .001). Participants receiving the decision critical communication gave higher ratings to the scientific evidence regarding the drug’s safety and effectiveness (Table 2). Sensitivity analyses found similar patterns among participants who had less formal education, reported that they would never use abortion medication, and completed the survey quickly.

**Table 1.** Self-Reported Characteristics of Final Sample (N = 311)

Characteristics^a^	Participants, No. (%)^b^
Age, mean (SD), y	31.7 (7.1)
Gender	
Genderqueer	2 (0.6)
Nonbinary	12 (3.9)
Woman	293 (94.2)
Prefer not to respond	4 (1.3)
Race and ethnicity^c^	
American Indian or Alaska Native	8 (2.6)
American Indian or Alaska Native (alone)	0
American Indian or Alaska Native (in combination with another response)	8 (2.6)
Asian	34 (10.9)
Asian (alone)	26 (8.4)
Asian (in combination)	8 (2.6)
Black or African American	61 (19.6)
Black or African American (alone)	47 (15.1)
Black or African American (in combination with another response)	14 (4.5)
Hispanic or Latino	38 (12.2)
Hispanic or Latino (alone)	18 (5.8)
Hispanic or Latino (in combination with another response)	20 (6.4)
Middle Eastern or North African	0
Native Hawaiian or Pacific Islander	0
White	202 (65.0)
White (alone)	176 (56.6)
White (in combination with another response)	26 (8.4)
Prefer not to answer	5 (1.6)
Education status	
High school or GED	96 (30.9)
Postgraduate degree	49 (15.8)
Technical/vocational college	42 (13.5)
Undergraduate degree	124 (39.9)
Abortion access in state of residence	
Full abortion access restrictions at the time of study (eg, Alabama, Florida)	125 (40.2)
No abortion restrictions at the time of study (eg, California, Maine)	183 (58.8)
Uncertain or limited abortion access restrictions at the time of study (eg, Iowa, Wisconsin)	3 (1.0)
Willingness to use mifepristone if needed	
Definitely yes	75 (24.1)
Probably yes	158 (50.8)
Probably not	40 (12.9)
Definitely not	9 (2.9)
Not applicable: “I do not support terminating a pregnancy”	29 (9.3)

Abbreviation: GED, General Educational Development.

^a^ All sample characteristics were directly queried of participants upon completion of the survey tasks.

^b^ Characteristics are distributed similarly across study groups, indicating successful randomization.

^c^ Race and ethnicity were queried within the same question, which allowed for multiple selection.

**Table 2.** Mann-Whitney Pairwise Group Comparisons

Measure	Mean (SD)	*P* value for pairwise comparisons^a^	*P* value for FDA vs decision critical^a^
**Usefulness of information (3 items)^b^**			
Readability			
Vendor	3.63 (0.99)	1 [Reference]	NA
FDA	4.21 (0.71)	<.001	>.99
Decision critical	4.25 (0.74)	<.001	
Supports decision-making			
Vendor	4.13 (0.78)	1 [Reference]	NA
FDA	3.95 (0.85)	.40	<.001
Decision critical	4.39 (0.67)	.03	
Supports proper use			
Vendor	4.33 (0.64)	1 [Reference]	NA
FDA	4.35 (0.63)	>.99	<.001
Decision critical	4.64 (0.59)	<.001	
**Comprehension (10 items)**^c^			
Knowledge common to all PMIs			
Vendor	5.50 (1.17)	1 [Reference]	NA
FDA	5.40 (0.76)	.63	<.001
Decision critical	6.34 (0.91)	<.001	
Quantified risk/benefit (decision critical) knowledge			
Vendor	0.39 (0.59)	1 [Reference]	NA
FDA	0.02 (0.14)	<.001	<.001
Decision critical	2.74 (0.52)	<.001	
**Perceptions of regulatory data (3 items)**^b^			
Strength of evidence			
Vendor	3.77 (0.86)	1 [Reference]	NA
FDA	3.65 (0.89)	.63	<.001
Decision critical	4.41 (0.71)	<.001	
Drug safety			
Vendor	3.76 (0.91)	1 [Reference]	NA
FDA	3.72 (0.90)	>.99	.002
Decision critical	4.08 (0.87)	.009	
Drug effectiveness			
Vendor	4.23 (0.81)	1 [Reference]	NA
FDA	4.00 (0.81)	.06	<.001
Decision critical	4.70 (0.66)	<.001	

Abbreviations: FDA, US Food and Drug Administration; NA, not applicable; PMI, Patient Medication Information.

^a^ The Bonferroni-adjusted significance level is .01, with *P* > .01 considered nonsignificant.

^b^ All items in this domain measured using a 5-point Likert scale.

^c^ Knowledge of information common to all PMIs is measured by number of correct responses out of 7 questions. Knowledge of quantified risk/benefit information measured by number of correct responses out of 3 questions.

## Conclusions

FDA’s proposed PMI was superior to the vendor communication in readability. Adding quantitative risk and benefit estimates increased participants’ comprehension, as well as their ratings of the quality of the scientific evidence and usefulness of the communication, without reducing gains in readability. Results were unrelated to education or willingness to take the drug. Limitations included recruiting paid volunteers, responding online, and the hypothetical nature of the evaluations, which lacked the cognitive and emotional engagement of actual choices. This study’s results suggest that succinct, readable, quantitative information can help patients understand drug risks and benefits. Such understanding may be especially important for drugs, such as mifepristone, that depend on patient self-management while being subject to widespread misinformation.

## Notes

Supplement 1.
Trial Protocol

Supplement 2. **eFigure 1.** Consolidated Standards of Reporting Trials (CONSORT) Flowchart for Trial Recruitment **eFigure 2.** Patient Medication Information Sheet Designed for Mifepristone [Mifeprex] According to FDA Guidelines **eFigure 3.** Decision Critical Patient Medication Information Sheet Designed for Mifepristone [Mifeprex] According to FDA Guidelines With Quantitative Risk and Benefit Information Added **eFigure 4.** Vendor Patient Medication Information Sheet for Mifepristone [Mifeprex]

Supplement 3.
Data Sharing Statement
