# Supplementary material for: Public Understanding of Risk and Benefit of Mifepristone: A Randomized Clinical Trial
Source: JAMA Netw Open. 2025 Feb 6;8(2):e2460236. doi: 10.1001/jamanetworkopen.2024.60236 (PMC11803473; doi:10.1001/jamanetworkopen.2024.60236)
Supplement: Supplement 1. — Trial Protocol [file jamanetwopen-e2460236-s001.pdf]

Table of Contents

1.0 Study Summary 2
2.0 Background 4
3.0 Objectives 4
4.0 Setting/Participants 4
5.0 Inclusion and Exclusion Criteria 5
6.0 Analyses and Data Management 5
7.0 Consent Process 8
8.0 Appendices 9
9.0 References 19

1.0 Study Summary

**Study Title**	Evaluating FDA's Proposed Patient Medication Information Handout: A Randomized Trial Measuring Perceived Usefulness and Comprehension
**Study Design**	**Allocation:** Randomized<br>**Interventional Model:** Parallel Assignment<br>**Interventional Model Description:** Participants will be randomized to receive either the one-page FDA-template PMI (FDA PMI), a modified template, which added quantitative information about drug risks and benefits (Decision Critical PMI), or the existing drug information insert (Vendor PMI – available at: https://www.fda.gov/media/164654/download).<br>Participants will complete the same survey questions measuring usefulness, comprehension of the information, and perceived strength of the regulatory evidence.<br>**Masking:** Single (Investigator)
**Primary Objective**	To compare FDA's new PMI template (*FDA PMI*) to 1) the current drug information insert (*Vendor PMI*) and 2) a decision science-informed revision (*Decision Critical PMI*)
**Research Intervention(s)/ Investigational Agent(s)**	**FDA template PMI**<br>Intervention is an information format viewed by participants. The patient medication information format was designed according to a proposed FDA template, which provides information about safe and effective use of a medication. No drug or device is included as part of this intervention.<br>**Decision Critical PMI**<br>Intervention is an information format viewed by participants. The patient medication information format was designed according to the proposed FDA template and modified, according to decision science principles, to include quantitative risk and benefit information, which provides information about safe and effective use of a medication. No drug or device is included as part of this intervention.<br>**Vendor PMI (Control)**<br>The intervention is an information format viewed by participants. This patient medication information format is the current patient medication information that is included with the drug. No drug or device is included as part of this intervention.
**Study Population**	US adults of reproductive age (18-45) who are assigned female at birth
**Sample Size**	330

**Study Duration for individual participants**	Participation is not anticipated to exceed 30 minutes
**Study Specific Abbreviations/ Definitions**	**BRF:** Benefit-Risk Framework<br>**FDA:** Food and Drug Administration<br>**PMI:** Prescription Medication Information<br>**RCT:** Randomized clinical trial

2.0 Background

In Fall 2023, FDA proposed amending its current prescription drug labeling regulations to facilitate patient decision-making.[1] It would require drug companies to provide patient medication information (PMI) in a new template. FDA proposed a one-page document that highlights the “essential information that patients need to know about the prescription drug product, including basic directions on how to use the product safely and effectively.” When evaluating drugs for approval, FDA uses a Benefit-Risk Framework (BRF).[2-4] The BRF considers risks, benefits, and alternatives for a drug. FDA references the BRF as guiding development of the new PMI, which largely aligns with the BRF. However, the current proposal does not contain information regarding a drug’s *benefit,* thereby omitting critical information for patients’ decision-making. FDA’s proposal also does not include any quantitative estimates of the medication’s risks or benefits, which decision science research has demonstrated that people utilize in their decision-making process. Additionally, the proposal does not require user testing of PMIs.

We aim to address these issues by (a) extending the proposed PMI to include benefit information and quantitative information about safety and effectiveness and (b) demonstrating a simple procedure for user testing. Using Mifeprex (generic: Mifepristone) as a case study, we demonstrate the procedure with a medication that is FDA-approved, safe, and effective medication for abortion and miscarriage management up to 10 weeks’ gestation.

3.0 Objectives

The primary objective of this study is to evaluate the effectiveness of prototypes displaying patient medical information (PMI) for prescription medications. Prototypes tested will display information for mifepristone, a medical abortion medication. We are interested in how the display of prescription medication information affects individuals’ comprehension and the best format to display this information.

The primary research questions are the following:

- How does the proposed one-page *FDA PMI* compare with the current drug information insert (*Vendor PMI*) in terms of usefulness and comprehension?
- How does the proposed one-page *FDA PMI* compare with a revision adding quantified risk/benefit information (*Decision Critical PMI*)?

4.0 Setting/Participants

Individuals will be recruited using the Prolific online survey recruitment platform, which pre-screens all individuals for pre-specific study eligibility criteria from a

nationally representative panel of research survey volunteers. Recruitment text is shown in Appendix 1. Participants who meet pre-specified eligibility criteria will be shown the recruitment text and can choose whether to continue to the survey and view the informed consent information. The Qualtrics survey platform will be used to administer the survey, where participants will be randomized, using the embedded Qualtrics randomization function, to view one of the 3 information formats in a 1:1:1 ratio. Participants will be paid $10 for completion of a survey that should take no longer than 30 minutes.

5.0 Inclusion and Exclusion Criteria

To be included in this study participants must self-identify as being biological capability of pregnancy but will not be excluded due to gender identity. Participants under age 18 or above age 45 will be excluded. Only those located within the US will be included. This information is collected by the Prolific platform and only individuals meeting these criteria will be shown the study recruitment materials.

The survey will include one attention check question (Appendix 2). Individuals who fail them will not be eligible for survey participation. Additionally, this study aims for 30% of the study sample to be comprised of individuals who are at/below high school/GED education level. Additional demographic information including gender, race, ethnicity, and residential zip code will be asked to characterize the participant sample but will not be considered as inclusion/exclusion criteria. Eligibility demographics will also be queried at the completion of the survey (age, sex) to confirm eligibility.

6.0 Analyses and Data Management

Intervention. Participants will be assigned randomly in parallel to one of the three following PMI formats:

FDA PMI

Patient medication information designed according to FDA template specifications. This template provides information about safe and effective use of a medication. No drug or device is included as part of this intervention.

Decision Critical PMI

Patient medication information designed according to FDA template and modified, according to decision science principles, to include quantified risk/benefit information. No drug or device is included as part of this intervention.

Vendor PMI (control)

This patient medication information format is the current standard of care patient medication information that is included with the drug. No drug or device is included as part of this intervention.

Primary outcome. Participants responses will be evaluated in the following domains:

<u>Usefulness of Information</u>

Readability

- Single question with a 5-point Likert scale response for "How easy or difficult was it to read the medication information guide about Mifeprex?" Minimum score is 1; Maximum score is 5. A higher score represents higher perceived readability.

Supports Decision Making

- Single question with a 5-point Likert scale response for "For someone who had <u>not yet decided</u> to use Mifeprex, how useful would the medication information guide be in helping them to make that decision?" Minimum score is 1. Maximum score is 5. A higher score represents higher perceived usefulness.

Supports Proper Use

- Single question with a 5-point Likert scale response for "If someone <u>had decided</u> to use Mifeprex, how useful would the medication information guide about [drug] be in helping them use the drug properly? " Minimum score is 1. Maximum score is 5. A higher score represents higher perceived usefulness.

<u>Comprehension</u>

- 10 newly constructed questions designed to measure the comprehension of the following information from the medication information guide: 1) Analysis of condition (1 question) 2) Risks (3 questions) 3) Risk Management (3 questions), and 5) Benefits (3 questions). Participants can score from 0-10 with 10 representing full comprehension of the information and 0 representing no comprehension of the information. Comprehension questions will be divided into two scores: *Knowledge Common to All PMIs* (Appendix 3 #1, 2, 6-10) and *Quantified Risk/Benefit Knowledge* (Appendix 3 #3-5).

Perceptions of Regulatory Data

Strength of Evidence

- Single question with a 5-point Likert scale response for "In your opinion, how strong is the *scientific evidence* that

Mifeprex, when taken correctly, helps patients end an early pregnancy?" Minimum score is 1; Maximum score is 5. A higher score represents stronger perceived evidence.

Drug Safety

- Single question with a 5-point Likert scale response for "In your opinion, how *safe* is Mifeprex, when taken correctly, for ending an early pregnancy?" Minimum score is 1. Maximum score is 5. A higher score represents higher perceived safety.

Drug Effectiveness

- Single question with a 5-point Likert scale response for "In your opinion, how *effective* is Mifeprex, when taken correctly, for ending an early pregnancy?" Minimum score is 1. Maximum score is 5. A higher score represents higher perceived effectiveness.

The full survey questionnaire can be found in Appendix 3.

Survey pretest. The PMI designs and survey questions were pretested with a convenience sample of the research team's social network (colleagues, family, friends). After revision, in response to this feedback, a pilot study of 30 participants was recruited on the Prolific platform to test the revised survey and inform power calculations for the study, which will use the same platform. No one who participated in the pilot will be eligible for inclusion in the main study.

Analyses. We will compare outcomes across the different PMI formats, first with a Kruskal-Wallis test for mean differences, followed by a Mann-Whitney test for pairwise comparisons. We will use a Bonferroni-correction to account for multiple comparisons, $\alpha = .01$ for ordinal variables. Additionally, we will conduct sensitivity analyses to assess changes in the primary outcomes (usefulness and comprehension): (1) including individuals who are excluded due to completing the survey too quickly (< 6.97 minutes, which represents the 10th percentile of completion time from the pilot data collection) and (2) excluding individuals who report not believing in using abortion medication under any circumstance (Appendix 3 #14), as they are unlikely to belong to the potential user population. To assess the distribution of the individuals participating in this study, optional questions regarding demographic information and the study participant's previous exposure Mifeprex (i.e., *Have you or someone you care about ever used the drug described in the medication guide?*) will be asked at the end of the survey. We will assess whether the Qualtrics randomization function was successful by checking the distribution of participants among the PMI groups. We should expect to see even distribution in group allocation, and approximately 30% of

each group to have at most a high school education. Other characteristics should be similarly distributed across study groups.

Based on response variance in our pilot study, a sample size of 100 participants per condition will yield statistical power with at least 85% chance of detecting a 0.5-point difference in comprehension (with a minimum score of 0 and maximum score of 7) between the *FDA PMI* and *Vendor PMI* group, assuming a two-sided $\alpha = .05$.

We hypothesize that the *FDA PMI* and *Decision Critical PMI* will be perceived as more readable and demonstrate greater comprehension than the *Vendor PMI*, as these formats are designed with readability and comprehension in mind. Additionally, we anticipate the *Decision Critical PMI* will be rated as more useful in supporting decision-making than the other two PMI formats. We do not anticipate differences in supporting proper drug use since supporting use is the primary goal of the *Vendor* and *FDA PMI* formats.

All analyses will be conducted using R Statistical Software (v4.3.2).

Ethical considerations. This study received expedited review and approval by Carnegie Mellon University’s Institutional Review Board (STUDY2023_00000449). The study protocol was registered on ClinicalTrials.gov (NCT06320808) and followed the Consolidated Standards of Reporting Trials (CONSORT) guidelines.

7.0 Consent Process

Participants will be provided with a consent page describing the purpose of the study and the procedure of the research. The informed consent page can be found in Appendix 4. Survey participation is entirely voluntary.

8.0 Appendices

Appendix 1. Recruitment language within the Prolific Platform.

This survey is being conducted by Dr. Baruch Fischhoff at Carnegie Mellon University, and Dr. Tamar Krishnamurti at the University of Pittsburgh. The purpose of the research is to better understand information provided with different medications. In this survey, you will be asked to read a consent form, view a document containing information about a medication, and answer a series of questions about the information you see on the document, and then answer demographic questions. This survey will take half an hour to complete, and you will be compensated $10.

Appendix 2. Study attention check.

Attention Check

We want to make sure that a human, and not a bot, is responding. **Please select the very last response (option 4) to the question below.** If you do not pass this attention check, you will not be eligible to continue and will be routed out of the survey. Following, your participation in the study, you will receive compensation within 1 week.

After reading the above paragraph, which is the correct option?
- ○ Option 1
- ○ Option 2
- ○ Option 3
- ○ Option 4

Appendix 3. Study survey questions and response options.

About this study

The U.S. Food & Drug Administration (FDA) will soon start providing people with new medication guides when they pick up prescription medication from the pharmacy. We are interested in your feedback on how well the guides work. The results from this survey will help us to make suggestions on how to improve the design of these guides. The next page shows an example of a prescription medication guide for a drug called Mifeprex.

Please read the guide and answer the questions that follow as best you can. You’ll be able to look at the guide when you answer the questions.

Take a moment to read through the medical guide shown below. You can download the medical guide from the link below and open it in a separate window if you would like to refer to it while you complete the survey questions. The guide will also be shown again on the following pages, beneath each survey question.

[Randomized PMI for Mifeprex is shown]

Now we will ask you some questions about Mifeprex. It is possible that some of these answers are not available on the guide you just saw. Please answer as best you can and, if you are not sure of an answer, it is ok to select or type "Unable to locate information." If you need it, the guide will be available to look at below each question.

1. What is Mifeprex used for?

- o To end an early pregnancy, up to 6 weeks
- o To end an early pregnancy, up to 10 weeks
- o To end a pregnancy in the first or second trimester
- o To end a pregnancy at any time before delivery
- o Unable to locate information

2. How should Mifeprex be taken?

- o Take one tablet of MIFEPREX and then take 2 more tablets of MIFEPREX 24-48 hours later
- o Take one tablet of MIFEPREX and then take 4 tablets of another drug called misoprostol 24-48 hours later
- o Take 3 tables of MIFEPREX at once
- o Take 3 tablets of MIFEPREX over a 48 hour period
- o Unable to locate information

3. What percentage of people taking Mifeprex experience a serious side effect?
Please enter a number. *If you cannot find the information, please skip this question and move onto the next.*

[ ]

4. Which statement is correct about how well Mifeprex works?

○ MIFEPREX when combined with misoprostol ends all pregnancies.
○ MIFEPREX, when combined with misoprostol, ends 97.4 % of pregnancies
○ MIFEPREX ends 100% of pregnancies without any complications.
○ MIFEPREX ends 90% of pregnancies, but the rate of birth defects is 10%
○ Unable to locate information

5. How many people participated in the clinical studies examining the effectiveness of Mifeprex? *Please enter a number. If you cannot find the information, please skip this question and move onto the next.*

[ ]

6. Who should not take Mifeprex? (select all that apply)

○ A woman seeking an abortion for an ectopic pregnancy
○ A woman who is 3 weeks' pregnant
○ A woman who is 10 weeks' pregnant
○ A woman who is currently using an IUD
○ Unable to locate information

7. Which of the following are common side effects of Mifeprex? (select all that apply)

☐ Headache
☐ Fever
☐ Hair loss
☐ Severe pelvic discomfort
☐ Unable to locate information

8. How can you contact the FDA to report side effects? (if you cannot locate this information, skip to the next question)

[ ]

9. Which of the following should a woman who has taken Mifeprex immediately call their healthcare provider about? (select all that apply)

☐ Bleeding or spotting for 14 days
☐ Developing a fever of 101 degrees that lasts all day
☐ Having mild abdominal pain
☐ Bleeding enough to soak through two pads every hour for three hours
☐ Unable to locate information

10. How long should a woman who has taken Mifeprex expect vaginal bleeding or spotting?

- ○ 24 to 48 hours
- ○ 9 to 16 days
- ○ 9 to 20 days
- ○ 10 weeks
- ○ Unable to locate information

The next set of questions ask your opinion about the drug, Mifeprex, that was described in the prescription medication guide you just saw.

11. In your opinion, how strong is the *scientific evidence* that Mifeprex, when taken correctly, helps patients end an early pregnancy?

- ○ Very weak
- ○ Weak
- ○ Neither weak nor strong
- ○ Strong
- ○ Very strong

12. In your opinion, how *safe* is Mifeprex, when taken correctly, for ending an early pregnancy?

- ○ Extremely safe
- ○ Somewhat safe
- ○ Neutral/Not sure
- ○ Somewhat unsafe
- ○ Extremely unsafe

13. In your opinion, how *effective* is Mifeprex, when taken correctly, for ending an early pregnancy?

- ○ Extremely effective
- ○ Somewhat effective
- ○ Neutral/Not sure
- ○ Somewhat ineffective
- ○ Very ineffective

14. If you had to end an early pregnancy, would you want to take Mifeprex to do so?

- ○ Definitely not
- ○ Probably not
- ○ Probably yes
- ○ Definitely yes
- ○ Not applicable – I do not support terminating a pregnancy

Thinking about the medication information sheet you saw about Mifeprex…

15. How *easy* or *difficult* was it to read the medication information guide about Mifeprex?

- o Very difficult
- o Difficult
- o Neither difficult or easy
- o Easy
- o Very easy

16. For someone who had not yet decided to use Mifeprex, how *useful* would the medication information guide be in helping them to make that decision?

- o Extremely useful
- o Useful
- o Neither useful or useless
- o Useless
- o Completely useless

17. If someone had decided to use Mifeprex, how *useful* would the medication information guide about Mifeprex be in helping them use the drug properly?

- o Extremely useful
- o Useful
- o Neither useful or useless
- o Useless
- o Completely useless

18. Is there any other information, not provided on the medication information guide, that would have been helpful to know?

This study showed information about a medication for abortion. As you may know, on June 24, 2022 the Supreme Court overturned *Roe v. Wade* This means that each U.S. state can decide its own policy on whether to ban or restrict abortion.

19. Which of the following best describes your feeling about the Supreme Court's overturning of *Roe v. Wade*?

- o It is taking our country very much in the right direction
- o It is taking our country somewhat in the right direction
- o I feel neutral/I don’t know
- o It is taking our country somewhat in the wrong direction
- o It is taking our country very much in the wrong direction

Lastly, we ask some demographic questions about you. We ask these questions to make sure we are surveying a diverse group of people.

20. What is your age?

21. What sex were you assigned at birth, meaning on your original birth certificate?

○ Male
○ Female
○ Prefer not to answer

22. Which of the following best describes your gender identity?

○ Male
○ Female
○ Non-binary
○ Prefer to Self-Describe (please specify)
○ Prefer not to answer

23. What is your race and ethnicity? Select all that apply

☐ American Indian or Alaska Native
☐ Asian
☐ Black or African American
☐ Hispanic or Latino
☐ Middle Eastern or North African
☐ Native Hawaiian or Pacific Islander
☐ White
☐ Prefer not to answer

24. Have you or someone you care about ever used the drug described in the medication guide?

○ Yes
○ No
○ I don't know
○ Prefer not to answer

25. What is your 5-digit zipcode?

26. If there is anything you would like to share that is not addressed in a survey question, we encourage you to add your comments here.

Appendix 4. Study consent.

Consent

This survey is part of a research study conducted by Dr. Baruch Fischhoff and Gianna White at Carnegie Mellon University and Dr. Tamar Krishnamurti at the University of Pittsburgh. The purpose of the research is to evaluate the best way to present the medical information included with prescription medications. You will see example information for a medication called Mifeprex and be asked to answer some questions about the information you see. This survey should take no more than 30 minutes to complete. You will be compensated $10 for your time and effort after you finish. There will be no cost to you if you participate in this study and you can stop at any time.

Participant Requirements

Participation in this study is limited to women located in the U.S between the ages of 18-45.

Risks & Benefits

The risks and discomfort associated with participation in this study are no greater than those ordinarily encountered in daily life or during other online activities. There is a potential risk of breach of confidentiality; to minimize this risk the data collected will only be accessible by approved study personnel and data will be stored on a password protected laptop or server. There may be no personal benefit from your participation in the study but the knowledge received might benefit other women in the future who use the medication described in the study. Although we will use the demographic information in our data analysis, any sharing of data with other researchers will be done so you will not be identified.

Future Use of Information

Your data will be used for research purposes only. The knowledge learned in this study may benefit other women in the future who use the medication described in the study.

Confidentiality

The data captured for the research does not include any personally identifiable information about you. Your IP address will not be captured.

Right to Ask Questions & Contact Information

If you have any questions about this study, you should feel free to ask them by contacting the Principal Investigator Gianna White, Department of Engineering and Public Policy, giannawhite@email.com. If you have questions later, desire additional information, or wish to withdraw your participation please contact the Principal Investigator by email, using the email address above.

If you have questions pertaining to your rights as a research participant; or to report concerns with this study, you should contact the Office of Research integrity and Compliance at Carnegie Mellon University. Email: irbreview@andrew.cmu.edu. Phone: 412-268-4721

Voluntary Participation

Your participation in this research is voluntary. You may discontinue participation at any time during the research activity. You may print a copy of this consent form for your records.

I have read and understand the information above and certify that I am eligible to participate in this research

○ Yes
○ No

I am 18 years or older and I want to participate in this research

○ Yes
○ No

E-signature (*you may use initials or a mark to indicate your signature*)

9.0 References

1. 88 Medication Guides: Patient Medication Information (FDA) 35694-35728 (May 31, 2023).
2. Food and Drug Administration. Structured Approach to Benefit-Risk Assessment in Drug Regulatory Decision-Making: Draft PDUFA V Implementation Plan Fiscal Years 2013–2017. https://www.fda.gov/media/84831/download
3. Food and Drug Administration. Benefit-Risk Assessment in Drug Regulatory Decision-Making: Draft PDUFA VI Implementation Plan (FY 2018–2022). https://www.fda.gov/media/112570/download
4. Lackey L, Thompson G, Eggers S. FDA's Benefit-Risk Framework for Human Drugs and Biologics: Role in Benefit-Risk Assessment and Analysis of Use for Drug Approvals. *Ther Innov Regul Sci*. Jan 2021;55(1):170-179. doi:10.1007/s43441-020-00203-6
