# Supplementary material for: Public Understanding of Risk and Benefit of Mifepristone: A Randomized Clinical Trial
Source: JAMA Netw Open. 2025 Feb 6;8(2):e2460236. doi: 10.1001/jamanetworkopen.2024.60236 (PMC11803473; doi:10.1001/jamanetworkopen.2024.60236)
Supplement: Supplement 2. — eFigure 1. Consolidated Standards of Reporting Trials (CONSORT) Flowchart for Trial Recruitment eFigure 2. Patient Medication Information Sheet Designed for Mifepristone [Mifeprex] According to FDA Guidelines eFigure 3. Decision Critical Patient Medication Information Sheet Designed for Mifepristone [Mifeprex] According to FDA Guidelines With Quantitative Risk and Benefit Information Added eFigure 4. Vendor Patient Medication Information Sheet for Mifepristone [Mifeprex] [file jamanetwopen-e2460236-s002.pdf]

Supplemental Online Content

Krishnamurti T, White G, Dewitt B, Mosley E, Fischhoff B. Public understanding of risk and benefit of mifepristone: a randomized clinical trial. *JAMA Netw Open*. 2025;8(2):e2460236. doi:10.1001/jamanetworkopen.2024.60236

eFigure 1. Consolidated Standards of Reporting Trials (CONSORT) Flowchart for Trial Recruitment

eFigure 2. Patient Medication Information Sheet Designed for Mifepristone [Mifeprex] According to FDA Guidelines

eFigure 3. Decision Critical Patient Medication Information Sheet Designed for Mifepristone [Mifeprex] According to FDA Guidelines With Quantitative Risk and Benefit Information Added

eFigure 4. Vendor Patient Medication Information Sheet for Mifepristone [Mifeprex]

This supplemental material has been provided by the authors to give readers additional information about their work.

© 2025 Krishnamurti T et al. *JAMA Network Open*.

eFigure 1. Consolidated Standards of Reporting Trials (CONSORT) flow chart for trial recruitment, enrollment, and analysis

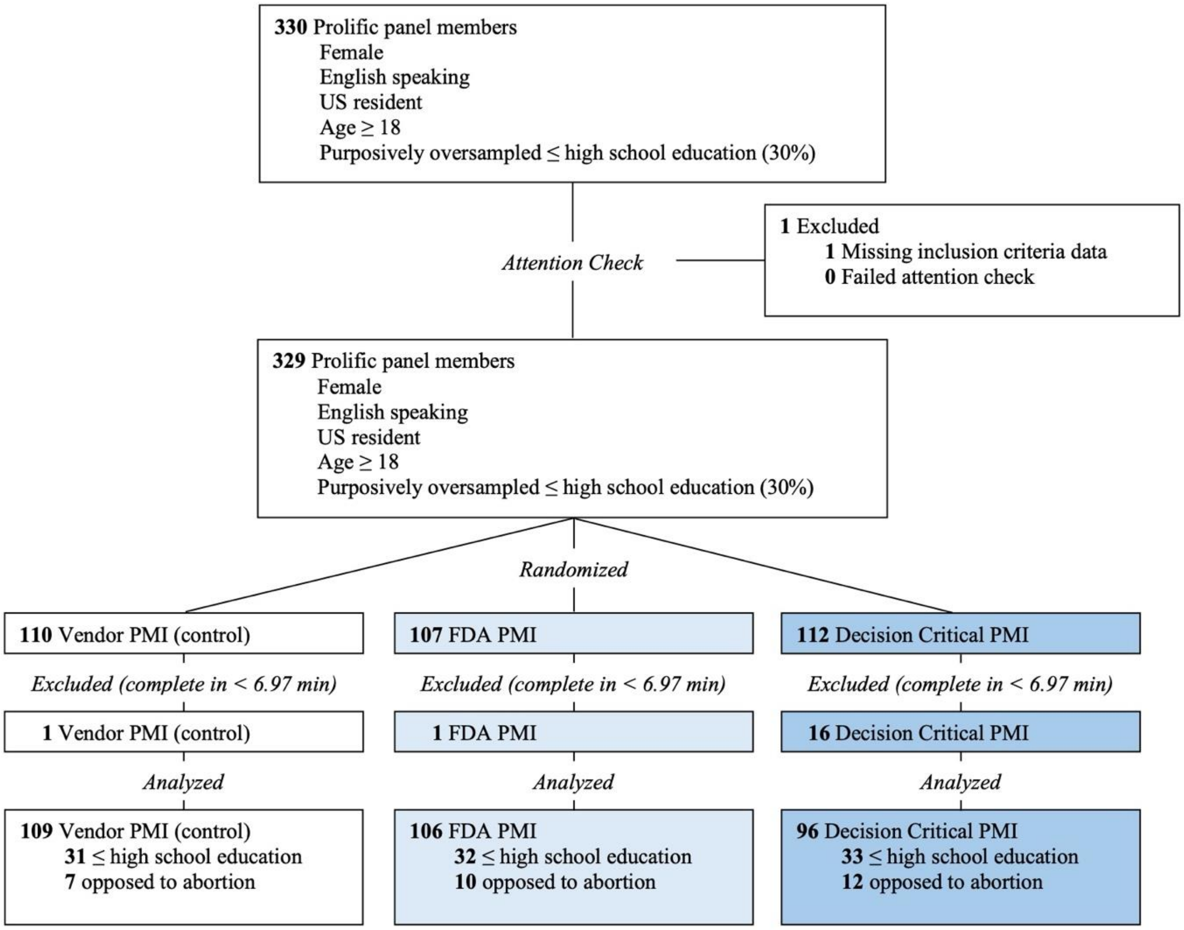

© 2025 Krishnamurti T et al. *JAMA Network Open*.

eFigure 2. Patient Medication Information Sheet designed for mifepristone [Mifeprex] according to FDA guidelines

PATIENT MEDICATION INFORMATION
MIFEPREX (Mifepristone) (MIF-e-PRIS-tone) tablets, 200 mg oral use

MIFEPREX is:

- used with another drug, misoprostol, to end an early pregnancy -- up to 10 weeks after the first day of your last menstrual period.

Important Safety Information

Warnings:

- Do not use MIFEPREX if you do not intend to end your pregnancy.
- Do not use MIFEPREX to end pregnancy beyond 10 weeks.
- Patients should expect to experience vaginal bleeding or spotting for about 9 to 16 days, with heavier than normal.
- menstrual period for the first few days.
- Bleeding and passing clots is expected. However, serious bleeding may require emergency care. Consider seeking medical attention if soaking through two or more sanitary pads in an hour for more than two hours, or if *any* bleeding is accompanied by dizziness, fatigue, palpitation, fainting, and/or a fever higher than 100.4 degrees F (38 degrees C).

Do Not Take

- Do not use MIFEPREX if you do not intend to end your pregnancy.
- Do not use MIFEPREX to end pregnancy beyond 10 weeks.
- Do not use MIFEPREX if you have or may have an ectopic pregnancy, routinely use steroids, or use an IUD.

Serious Side Effects:

Call your health care provider at once if you have:

- a fever higher than 100.4 degrees F (38 degrees C) lasting longer than 4 hours
- severe abdominal pain or pelvic tenderness
- prolonged heavy vaginal bleeding (enough blood to soak 2 or more sanitary pads in an hour for more than 2 hours

Tell Your Healthcare Provider Before Taking:

- If you cannot arrange a follow-up appointment within 7 to 14 days
- If you are breastfeeding, MIFEPREX can pass into your breast milk, with unknown effects.
- If you are taking other medicines, including prescription and over-the-counter drugs, vitamins, and herbal supplements.

Common Side Effects

The most common reported side effects in adults were:

- Fever
- Chills
- Weakness
- Headache
- Dizziness
- Nausea
- Vomiting
- Diarrhea

This is not a complete list of side effects. Call your health care provider if you have questions about side effects. You may report side effects to FDA at 1-800-FDA-1088.

Directions for Use

Use MIFEPREX exactly as prescribed. Follow all directions on your prescription label and read all medication guides or instruction sheets. Take 1 MIFEPREX tablet orally on Day 1. After 24 to 48 hours, take 4 misoprostol tablets by placing 2 tablets in each cheek pouch for 30 minutes, then swallow with water. Expect common side effects. Follow up with a healthcare provider 7 to 14 days later to ensure pregnancy has passed. If not, discuss further options with your healthcare provider.

Manufactured by: Danco Laboratories, LLC, P.O. Box 4816, New York, NY 10185

The content of this Patient Medication Information has been approved by the U.S. Food and Drug Administration Revised: 10/2023

© 2025 Krishnamurti T et al. *JAMA Network Open*.

eFigure 3. Decision Critical Patient Medication Information Sheet designed for mifepristone [Mifeprex] according to FDA guidelines with quantitative risk and benefit information added

PATIENT MEDICATION INFORMATION

MIFEPREX (Mifepristone) (MIF-e-PRIS-tone) tablets, 200 mg oral use

MIFEPREX is:

• used to end an early pregnancy -- up to 10 weeks after the first day of your last menstrual period.

How does this drug work?

• MIFEPREX blocks a hormone needed to maintain a pregnancy. It is a single tablet. 24 to 48 hours after taking MIFEPREX, patients take four tablets of another drug called misoprostol. Misoprostol pushes the pregnancy out of the uterus with contractions and bleeding. To end an early pregnancy, MIFEPREX and misoprostol must both be taken.

Who might consider taking it?

Individuals seeking to end an early pregnancy.

How well does MIFEPREX work?

• In clinical studies with 16,794 U.S. women, MIFEPREX ended 97.4% of pregnancies, with 1.9% needing a medical procedure, due to persistent or heavy bleeding, patient request, or incomplete expulsion. It did not end 0.7% of pregnancies.

What to expect?

• The uterus should begin to pass the pregnancy within 2 to 24 hours after taking MIFEPREX and misoprostol. When the pregnancy is passed, you will have bleeding and cramping that is likely to be heavier than your usual menstrual period.

• Patients should expect to vaginal bleeding or spotting for about 9 to 16 days, with heavier than normal menstrual period for the first few days.

• Bleeding and passing clots are expected. However, serious bleeding may require emergency care.

• Consider seeking medical attention if soaking through two or more sanitary pads in an hour for more than two hours, or if *any* bleeding is accompanied by dizziness, fatigue, palpitation, fainting, and/or a fever higher than 100.4 ° F (38 ° C).

What other choices are there?

• Methotrexate with Misoprostol: Another form of medication abortion. It works similarly to MIFEPREX.

• A Procedural Abortion: an in-clinical medical procedure to terminate a pregnancy.

Important Safety Information

Warnings:

• Do not use MIFEPREX if you do not intend to end your pregnancy.

• Do not use MIFEPREX to end pregnancy beyond 10 weeks.

• Do not use MIFEPREX if you have or may have an ectopic pregnancy, chronic use of steroids, or use an IUD.

Serious Side Effects:

MIFEPREX is available only through a restricted program under a REMS called the Mifepristone REMS Program, because of the risks of serious complications. 1% of users experience serious side effects of infections or bleeding that could be fatal. Call your health care provider at once if you have:

• a fever higher than 100.4 degrees F (38 degrees C) lasting longer than 4 hours

• severe abdominal pain or pelvic tenderness

• prolonged heavy vaginal bleeding (enough blood to soak 2 or more sanitary pads in an hour for more than 2 hours)

Tell Your Healthcare Provider Before Taking:

• If you cannot arrange a follow-up appointment within 7 to 14 days

• If you are breastfeeding, because MIFEPREX can pass into your breast milk, with unknown effects.

• If you are taking other medicines, including prescription and over-the-counter drugs, vitamins, and herbal supplements.

Common Side Effects

The most common side effects were: fever or chills (48% of patients), weakness (about 57%), headache (about 43%), dizziness (about 40%), nausea (about 63%), vomiting (about 43%), diarrhea (about 30%).

This is not a complete list of side effects. Call your health care provider if you have questions about side effects. You may report side effects to FDA at 1-800-FDA-1088, so that they can learn about peoples' experience taking the drug.

Directions for Use

Use MIFEPREX exactly as prescribed. Follow all directions on your prescription label and read all medication guides or instruction sheets. Take 1 MIFEPREX tablet orally on Day 1. After 24 to 48 hours, take 4 misoprostol tablets by placing 2 tablets in each cheek pouch for 30 minutes, then swallow with water. Expect common side effects. Follow up with a healthcare provider 7 to 14 days later to ensure pregnancy has passed. If not, discuss further options with your healthcare provider.

Manufactured by: Danco Laboratories, LLC, P.O. Box 4816, New York, NY 10185

The content of this Patient Medication Information has been approved by the U.S. Food and Drug Administration Revised: 10/2023

© 2025 Krishnamurti T et al. *JAMA Network Open*.

eFigure 4. Vendor Patient Medication Information Sheet for mifepristone [Mifeprex]

The five-page medication guide used as for the *Vendor PMI* (control) was approved by the U.S. Food and Drug Administration on 01/2023 and accessed by the research team from the following source: https://www.fda.gov/media/164654/download

© 2025 Krishnamurti T et al. *JAMA Network Open*.
