# Supplementary material for: Public Understanding of Risk and Benefit of Mifepristone: A Randomized Clinical Trial
Source: JAMA Netw Open. 2025 Feb 6;8(2):e2460236. doi: 10.1001/jamanetworkopen.2024.60236 (PMC11803473; doi:10.1001/jamanetworkopen.2024.60236)
Supplement: Supplement 3. — Data Sharing Statement [file jamanetwopen-e2460236-s003.pdf]

Data Sharing Statement

Krishnamurti. Public Understanding of Risk and Benefit of Mifepristone. *JAMA Netw Open*. Published February 06, 2025. doi:10.1001/jamanetworkopen.2024.60236

Data
Data available: Yes
Data types: Deidentified participant data
How to access data: Data can be made available upon request from tamark@pitt.edu
When available: With publication

Supporting Documents
Document types: None

Additional Information
Who can access the data: researchers whose proposed use of the data has been approved
Types of analyses: for non-commercial research purposes upon institutional approval of request
Mechanisms of data availability: upon institutional approval of request
